# Supplementary material for: Revisiting the link between childhood adversity and stress-sensitive brain regions in psychosis and bipolar disorder: a systematic review and meta-analysis
Source: Transl Psychiatry. 2026 Sep 19;16:539. doi: 10.1038/s41398-026-04457-w (PMC13650146; doi:10.1038/s41398-026-04457-w)
Supplement: Supplementary file 1 [file 41398_2026_4457_MOESM1_ESM.pdf]

Supplementary material

Supplementary Table 1. PRISMA Checklist

Section/Topic	#	Checklist Item	Page
**TITLE**			
Title	1	Identify the report as a systematic review, meta-analysis or both.	1
**ABSTRACT**			
Structured Summary	2	Provide a structured summary including as applicable: background; objectives; data sources; study eligibility criteria, participants, and interventions; study appraisal and synthesis methods; results; limitations; conclusions and implications of key findings; systematic review and registration number.	2
**INTRODUCTION**			
Rationale	3	Describe the rationale for the review in the context of existing knowledge.	3-4
Objectives	4	Provide an explicit statement of objective(s) or question(s) the review addresses with reference to participants, interventions, comparison, outcomes, and study design (PICOS).	5
**METHODS**			
Eligibility Criteria	5	Specify the inclusion and exclusion criteria for the review and how studies were grouped for syntheses.	6-7
Information Sources	6	Specify all databases, registers, websites, organisations, reference lists and other sources searched or consulted to identify studies. Specify the date when each source was last searched or consulted.	5

Supplementary material

Search Strategy	7	Present full electronic search strategy for at least one database, including any limits used, such that it could be repeated.	5 and Supplementary material (SM)
Selection Process	8	State the process for selecting studies (i.e. screening, eligibility, included in systematic review, and if applicable, included in meta-analysis).	6-7
Data Collection Process	9	Specify the methods used to collect data from reports, including how many reviewers collected data from each report, whether they worked independently, any processes for obtaining or confirming data from study investigators, and if applicable, details of automation tools used in the process.	6
Data Items	10a	List and define all outcomes for which data were sought. Specify whether all results that were compatible with each outcome domain in each study were sought (e.g. for all measures, time points, analyses), and if not, the methods used to decide which results to collect.	6 and SM
	10b	List and define all other variables for which data were sought (e.g. participant and intervention characteristics, funding sources). Describe any assumptions made about any missing or unclear information.	6 and SM
Study Risk of Bias Assessment	11	Specify the methods used to assess risk of bias in the included studies, including details of the tools used, how many reviewers assessed each study and whether they worked independently, and if applicable, details of automation tools used in the process.	7
Effect Measures	12	Specify for each outcome the effect measures (e.g. risk ratio, mean difference) used in the synthesis or presentation of results.	-

Supplementary material

Synthesis Methods	13a	Describe the processes used to decide which studies were eligible for each synthesis, (e.g. tabulating the study intervention characteristics and comparing against the planned groups for synthesis).	5-7
	13b	Describe any methods required to prepare the data for presentation or synthesis, such as handling of missing summary statistics, or data conversion.	5-7
	13c	Describe any methods used to tabulate or visually display results of individual studies and synthesis.	5-7
	13d	Describe any methods used to synthesise results and provide rationale for the choices. If meta-analysis was performed, describe the models, methods to identify the presence and extent of statistical heterogeneity, and software packages used.	5-7
	13e	Describe any methods used to explore possible causes of heterogeneity among study results (e.g. subgroup analyses, meta-regression).	-
	13f	Describe any sensitivity analyses conducted to assess robustness of the synthesised results.	-
Reporting Bias Assessment	14	Describe any methods used to assess risk of bias due to missing results in a synthesis (arising from reporting biases).	-
Certainty Assessment	15	Describe any methods used to assess certainty (or confidence) in the body of evidence for an outcome.	5-7
**RESULTS**			
Study Selection	16a	Describe the results of the search and selection process, from the number of records identified in the search to the number of studies included in the review, ideally using a flow diagram.	7-8

Supplementary material

	16b	Cite studies that might appear to meet inclusion criteria, but which were excluded, and explain why they were excluded.	7-9
Study Characteristics	17	Cite each included study and present its characteristics.	7-9
Risk of Bias In Studies	18	Present assessment of risk of bias for each included study.	8
Results in Individual Studies	19	For all outcomes, present, for each study: (a) summary statistics for each group (where appropriate) and (b) an effect estimates and its precision (e.g. confidence/credible intervals), ideally using structured tables and plots.	7-9
Results of Synthesis	20a	For each synthesis, briefly summarise the characteristics and risk of bias among contributed studies.	8
	20b	Present results of all statistical syntheses conducted. If meta-analyses was done, present for each the summary estimate and its precision (e.g. confidence/credible interval) and measures of statistical heterogeneity. If comparing groups, describe the direction of the effect.	-
	20c	Present results of all investigations of possible causes of heterogeneity among study results.	-
	20d	Present results of all sensitivity analyses conducted to assess the robustness of the synthesised results.	-
Reporting Bias	21	Present assessments of risk of bias due to missing results (arising from reporting biases) for each synthesis assessed.	7-9
Certainty of Evidence	22	Present assessments of certainty (or confidence) in the body of evidence for each outcome assessed.	-
**DISCUSSION**			

Supplementary material

Discussion	23a	Provide a general interpretation of the results in the context of other evidence.	10
	23b	Discuss any limitations of the evidence included in the review.	15-17
	23c	Discuss any limitation of the review processes used.	15-17
	23d	Discuss implications of the results for practice, policy, and future research.	17-18
**OTHER INFORMATION**			
Registration and protocol	24a	Describe sources of funding for the systematic review and other support; role of funders for the systematic review.	MRC
	24b	Indicate where the review protocol can be accessed, or state that a protocol was not prepared.	Prospero. CRD42022351133
	24c	Describe and explain any amendments to information provided at registration or in the protocol.	NA
Support	25	Describe sources of financial or non-financial support for the review, and the role of the funders or sponsors in the review.	NA
Competing Interests	26	Declare any competing interests of review authors.	NA
Availability of Data, Code, and Other Materials	27	Report which of the following are publicly available and where they can be found: template data collection forms; data extracted from included studies; data used for all analyses; analytic code; any other materials used in the review.	NA

Note. Adapted from the PRISMA 2020 Statement (Page et al., 2021).

Supplementary material

Supplementary Table 2. Full list of search terms used in the literature search

---

The search term used on OVID:
Mesh terms (starting with “exp” and key words (ending with “mp”) related to CA
exp Sex Offenses/
exp Physical Abuse/
exp Child Abuse/
exp Bullying/
exp Sexual Abuse/
exp Physical Abuse/
exp Emotional Abuse/
exp Child Abuse/
exp CHILD NEGLECT/
exp Emotional Trauma/
exp BULLYING/
exp Abandonment/
exp RAPE/
exp Domestic Violence/
exp Sexual Harassment/
exp Rape/
exp Domestic Violence/
exp Sexual Harassment/
exp sexual abuse/
exp physical abuse/
exp emotional abuse/
exp neglect/
exp child abuse/
exp bullying/
exp sexual bullying/

Supplementary material

exp rape/
exp domestic violence/
exp psychotrauma/
exp early life stress/
exp sexual harassment/
(separat* adj5 parent*).mp. [mp=ti, ab, hw, tn, ot, dm, mf, dv, kf, fx, dq, nm, ox, px, rx, ui, sy, tc, id, tm]
victimi*.mp.
(advers* adj5 experienc*).mp. [mp=ti, ab, hw, tn, ot, dm, mf, dv, kf, fx, dq, nm, ox, px, rx, ui, sy, tc, id, tm]
adversit*.mp.
emotional abuse.mp. or exp emotional abuse/
psychological abuse.mp.
neglect.mp.
bully*.mp.
bullied*.mp.
parental loss.mp. or exp parental deprivation/
(Childhood adj5 trauma).mp. [mp=ti, ab, hw, tn, ot, dm, mf, dv, kf, fx, dq, nm, ox, px, rx, ui, sy, tc, id, tm]
(abandon adj10 parent*).mp. [mp=ti, ab, hw, tn, ot, dm, mf, dv, kf, fx, dq, nm, ox, px, rx, ui, sy, tc, id, tm]
maltreat*.mp.
(Parent adj5 loss).mp. [mp=ti, ab, hw, tn, ot, dm, mf, dv, kf, fx, dq, nm, ox, px, rx, ui, sy, tc, id, tm]
(Childhood adj5 maltreat*).mp. [mp=ti, ab, hw, tn, ot, dm, mf, dv, kf, fx, dq, nm, ox, px, rx, ui, sy, tc, id, tm]
early adversity.mp.
being taken into care.mp.
early life stress.mp. or exp early life stress/
exp rape/ or rape.mp.
domestic violence.mp.
sexual harassment.mp.
exp psychotrauma/
sexual abuse.mp.
physical abuse.mp.
Mesh terms (starting with “exp” and key words (ending with “mp”) related to Psychotic disorders and bipolar disorders.

Supplementary material

exp psychosis/
exp schizophrenia/
exp schizoaffective psychosis/
exp Psychotic Disorders/
exp Schizophrenia/
exp PSYCHOSIS/
exp Acute Psychosis/
exp Affective Psychosis/
exp SCHIZOPHRENIA/
exp SCHIZOAFFECTIVE DISORDER/
psychot*.mp.
schizophr*.mp.
schizoaf*.mp. [mp=ti, ab, hw, tn, ot, dm, mf, dv, kf, fx, dq, nm, ox, px, rx, ui, sy, tc, id, tm]
hallucinat*.mp.
delusion*.mp.
exp bipolar disorder/
exp manic psychosis/
exp mania/
exp bipolar mania/
exp Bipolar Disorder/
exp Bipolar I Disorder/
exp Bipolar II Disorder/
Bipolar disorder.mp.
mania.mp.
maniaco depressive.mp.
exp Hypomania/
hypomania.mp.
Mesh terms (starting with “exp” and key words (ending with “mp”) related to brain imaging data
exp Neuroimaging/
exp Magnetic Resonance Imaging/

Supplementary material

exp White Matter/
exp neuroimaging/
exp imaging/
exp diffusion tensor imaging/
exp nuclear magnetic resonance/
brain scan.mp.
imaging data.mp.
morphometry.mp.
DTI.mp.
neuroimaging.mp.
structural imaging.mp.
exp Gray Matter/
gray matter.mp.
brain volume.mp.
brain size.mp.
brain structure.mp.
exp Magnetic Resonance Imaging/
MRI.mp.
grey matter.mp.

Supplementary material

Supplementary table 3. Diagnostic codes

Diagnosis	Code used within ICD-10	Code used within DSM-IV
**Schizophrenia**	F20	295.10/295.20/295.30/295.60/295.90
**Brief psychotic disorder**	F23	-
**Schizophreniform disorder**	F20.81	Schizophreniform disorder
**Schizoaffective disorder**	F25.0	295.70
**Major depressive disorder with psychotic features**	F33.0	296.24/296.34
**Psychosis not otherwise specified**	F29	298.9
**Bipolar disorder type II**	F31.81	296.89
**Bipolar disorder type I (bipolar affective disorder)**	F31.1	296.xx (all subtypes) including F31.2 severe with psychotic features

Supplementary material

Supplementary Table 4. Newcastle Ottawa Scale

**Newcastle Ottawa Scale (adapted from the cohort studies NOS scale)**<br>Note: A study can be awarded a maximum of one star for each numbered item within the Selection and Outcome categories. A maximum of two stars can be given for Comparability.
**Selection**<br>1) Representativeness of the exposed cohort (b)<br>a) truly representative of the average individuals with psychosis or bipolar in the community (Really when ALL cases were part of the study - birth cohorts, totally representative) *<br>b) somewhat representative of the average individuals with psychosis or attenuated psychotic symptoms in the community *<br>c) selected group of users eg nurses, volunteers (not applicable for us because of selection criteria excludes these two options)<br>d) no description of the derivation of the cohort (not applicable for us because of selection criteria excludes these two options)<br>2) Selection of the non-exposed cohort (a)<br>a) drawn from the same community as the exposed cohort * (always a, but check that the trauma and non-trauma are extracted from the same cohort – we are not picking traumatised from Norway and non traumatised from Lausanne)<br>b) drawn from a different source (if they were taking this from a different population)<br>c) no description of the derivation of the non-exposed cohort (not applicable for us because of selection criteria excludes these two options)<br>3) Ascertainment of exposure (c) (between b and c for us)<br>a) secure record*<br>b) structured interview*<br>c) written self-report* (this has been modified: star included here given the common use of self-reports in the field of adversity in psychosis)<br>d) no description (these are excluded)<br>4) Demonstration that outcome of interest was not present at start of study (is the problem already there (the brain abnormality) when the patients have been assessed for the first time? For example, covid infection in pregnant women and we want to see if later children develop autism, the study starts at birth (when the outcome of interest is not present, so it would be “yes”, however for most of our clinical samples the problem (low functioning), is already there, so it will likely be “no” but exclude that samples come from prospective studies from birth).<br>a) yes *<br>b) no

Supplementary material

Comparability (count 2 stars)

1) Comparability of cohorts on the basis of the design or analysis

a) study controls for confounders * (one star: studies providing adjusted and unadjusted results and/or adjusting by covariates such as age, gender, race, education – often in linear regressions, logistic regressions etc. Correlation, t- test, qui square does not adjust – check statistics; b) study includes a sample of N>=100) *

Outcome

1) Assessment of outcome

a) independent blind assessment and using validated advances programs * (in imaging data usually this is done by technicians that don't know the traumatic history, programs like Freesurfer)

b) record linkage or formal interview of symptoms (doe sot apply)*

c) self-report

d) no description

2) Was follow-up long enough for outcomes to occur

a) yes * (this will only apply for birth cohorts, for cross-sectional studies does not apply so no star)

b) no

3) Adequacy of follow up of cohorts (it could be that some that drop out were treatment resistant) (>20%)

a) complete follow up (no drop outs) - all subjects accounted for *

b) subjects lost to follow up unlikely to introduce bias - small number lost < 20 % *

c) follow up rate < 80% and no description of those lost

d) no statement/ no follow up (cross sectional studies)

Supplementary material

Supplementary Table 4a. Newcastle Ottawa Scale (NOS) Quality Assessment for the included studies

	**Psychosis sample**											
**Author**	**Selection**					**Comparability**		**Outcome**				**Total/ Quality Classification**
	1. Represen-tativeness of exposed cohort	2. Selection of the non-exposed cohort	3. Ascertain-ment of exposure	4. Outcome of interest was not present at start of study	Domain Score (out of 4)	1. Compara-bility of cohorts on the basis of the design or analysis	Domain Score (out of 2)	1. Assess-ment of outcome	2. Ade-quate length of follow-up	3. Adequate follow-up of cohorts	Domain Score (out of 3)	
Aas et al., (2012)	*	*	*		3/4	*	1/2	*			1/3	5/ Fair
Alameda et al., (2018)	*	*	*		3/4	**	2/2	*			1/3	6/ Good
Armio et al., (2020)	*	*	*		3/4	*	1/2	*			1/3	5/ Fair
Begemann et al., (2021)	*	*	*		3/4	**	2/2	*			1/3	6/ Good
Benedetti et al., (2011)	*	*	*		3/4	*	1/2	*			1/3	5/ Fair
Cancel et al., (2015)	*	*	*		3/4	*	1/2	*			1/3	5/ Fair
Cao et al. (2023)	*	*	*		3/4	**	1/2	*			1/3	6/ Good
Colic et al. (2024)	*	*	*		3/4	**	2/2	*			1/3	6/ Good
Del Re et al., (2023)	*	*	*		3/4	**	2/2	*			1/3	6/ Good
Duarte et al., (2016)	*	*	*		3/4	*	1/2	*			1/3	5/ Fair

Supplementary material

Elvsaashagen et al., (2013)	*	*	*		3/4	*	1/2	*			1/3	5/ Fair
Du Plessis et al., (2020)	*	*	*		3/4	*	1/2	*			1/3	5/ Fair
Fan et al., (2022)	*	*	*		3/4	**	2/2	*			1/3	6/ Good
Frissen et al., (2018)	*	*	*		3/4	*	1/2	*			1/3	5/ Fair
Harmata et al., (2023)	*	*	*		3/4	**	2/2	*			1/3	6/ Good
Hernaus et al., (2014)	*	*	*		3/4	*	1/2	*			1/3	5/ Fair
Hoffmann et al., (2018)	*	*	*		3/4	**	2/2	*			1/3	6/ Good
Hoy et al., (2012)	*	*	*		3/4	*	1/2	*			1/3	5/ Fair
Janiri et al., (2017)	*	*	*		3/4	**	22	*			1/3	6/ Good
Janiri et al., (2017)	*	*	*		3/4	**	2/2	*			1/3	6/ Good
Millman et al., (2022)	*	*	*		3/4	**	2/2	*			1/3	6/ Good
Poletti et al., (2014)	*	*	*		3/4	*	1/2	*			1/3	5/ Fair
Quidé et al., (2021)	*	*	*		3/4	**	2/2	*			1/3	6/ Good
Rokita et al., 2020	*	*	*		3/4	**	1/2	*			1/3	5/ Fair
Ruby et al., (2017)	*	*	*		3/4		0/2	*			1/3	4/ Poor
Sheffield et al., (2013)	*	*	*		3/4	*	1/2	*			1/3	5/ Fair
Song et al., (2020)	*	*	*		3/4	*	1/2	*			1/3	5/ Fair

Supplementary material

Souza-Queiroz et al., (2016)	*	*	*		3/4	*	1/2	*			1/3	5/ Fair
Tseng et al., (2021)	*	*	*		3/4	*	1/2	*			1/3	5/ Fair

Supplementary material

Supplementary Table 5. Study Characteristics and Neuroimaging Outcome Parameters

Author (Year), Country	Diagnosis specification	Diagnosis Measure	CA Variable type	Regions reported in relation to CA	Details on the association of CA on Brain Measurements	Covariates	Inclusion meta-analysis
**Aas et al. (2012)**	FEP	ICD-10	CECA.Q<br>Composite<br><br>(categorical)	1.Hippocampus<br>2. Amygdala	**1**. NSA (total, left, right)<br>**2**. r = - .40, *p* = - .006<br>Right amygdala: f = 4.80; p = .01<br>Left amygdala: f=4.90; p=.01	Age<br>Sex<br>ICV	yes
**Alameda et al. (2018)**	FEP	DSM-IV	Psychiatric Interview<br>Composite<br>(binary)	1. Hippocampus<br>2. Amygdala	**1**. NSA Mean and right hippocampus<br>Left hippocampus: d = 0.51, p = 0.046<br>**2.** NSA mean, right and left amygdala	-	no
**Armio et al. (2020)**	FEP	DSM-IV	TAD<br>Composite<br><br>(continuous)	Amygdala subnuclei:<br>1. Lateral nucleus<br>2. Basal nucleus<br>3. Cental nucleus<br>4. Accessory basal nucleus<br>5. Corticoamygdaloid transition area	**1.** β = -1.45, t (62) = - 2.852, *p* = .006<br>**2.** β = -0.86, 7 (62) = -2.350, p = 0.022<br>3. NSA<br>4.NSA<br>5. NSA	Age<br>Sex<br>ICV<br>FDR	no
**Begemann et al. (2021)**	SCZ<br>BD-I	DSM-IV	CTQ<br>Composite &<br>Subtypes: SA, PA, EA, PN, EN<br>(binary and continuous)	1.Hippocampus<br>2.Amygdala<br>**68 regions grouped into cortical lobes:**<br>3.Frontal Lobe<br>4.Temporal lobe<br>5.Cingulate/insular lobe<br>6.Subcortical lobe<br>7.Parietal lobe<br>8.Occipital lobe<br>9.Total GMV	**1.** NSA (left and right)<br>**2**. NSA (left and right)<br>**3**. Right medialorbitofrontal region (β = -0.107, p = 0.002)<br>Right paracentral region (β = -0.088, p = 0.018)<br>Right superior frontal area (β = -0.058, p = 0.027)<br>Left precentral regions (β = -0.057, p = 0.0498)<br>**4-9.** NSA<br>NB. Reported results are only significant in the total sample, not significants in PD only.	Age<br>Sex<br>Group<br>Brain volume<br>FDR<br>Type of Scan<br>Medication	no

Supplementary material

**Benedetti et al. (2011)**	SCZ	DSM-IV (SCID-I) SCZ = 100 %	RFQ<br><br>(binary)	1.PFC<br>2.ACC<br>3. Hippocampus<br>4. Amygdala	1. p<0.001<br>2. p<0.001<br>3. NSA<br>4. NSA	Medication<br>FDR correction	no
**Cancel et al. (2015)**	SCZ	DSM-IV SCZ = 100%	CTQ Composite & Subtypes : EN, PN, EA, SA<br><br>(continuous)	1. Total GMV<br>2. Right DLPFC GMV<br>3. Left DLPFC GMV<br>4. Right Inferior Frontal Gyrus	**1**.EN : β = -.47, *p* = .005 ; PA, PN, SA : NSA<br>**2**.EN: Right (k = 1113 voxels, $P_{cluster}$ = 0.003, FWE-corrected, P_{voxel}<0.002 FWE-uncorrected, z-$score_{voxels}$ = 4.33 at [40;38;25])<br>**3.**EN: NSA<br>**4.** EN: NSA	Age<br>Gender<br>DUI<br>Parents educational<br>Medication use<br>Bonferroni correction	no
**Cao et al. (2024)**	BD-II	DSM-V 100 = BD-II	CTQ (total, PN, EN, SA, EA	1.Right MFG<br>2.Right orbital MFG	1.SA, r = -0.348, q<0.01<br>2.PN, r=0.254, q = .03	Age<br>Sex<br>Education level<br>Medication<br>FDR correction	no
**Colic et al. (2022)**	BD	CTQ composite & subtypes : SA, PA, EA, PN, EN (continuous)	sMRI (3T)<br>ROI and whole-brain<br>Automated segmentation	1.Left hippocampus<br>2.Right hippocampus<br>3.Prefrontal cortex regions surface area (superior frontal, rostral middle frontal, frontal pole, rostral anterior congulate, medial orbitofrontal)	1.B = -7.41, p = .03 (SA, in women)<br>2.NSA<br>3.Frontal pole surface (CTQ composite, EN, EA, PN in men)<br>Right superior frontal surface area (EN, PA, SA in men)	Age<br>ICV<br>FDR correction	no
**Del Re et al. (2023)**	SCZ	DSM-IV-TR	CTQ Composite<br><br>(continuous)	1.Hippocampus	**1.**Hippocampus<br>Right anterior hippocampus (r = -0.11, p = 0.011)<br>Right posterior hippocampus (r = -0.12, p = 0.003)<br>Left anterior hippocampus NSA<br>Left posterior hippocampus (r =-0.14, p = 0.0004)	Age<br>Sex<br>ICV<br>Race<br>MRI site<br>FDR	no

Supplementary material

**Duarte et al. (2016)**	BD-I	MINI-Plus	CTQ composite, Subtypes (PN, EN, PA) (continuous)	GMV:<br>1.Right DLPFC<br>2.Bilateral Thalamus	**1.** CTQ Total : Z=4.37, voxel= 149, p=.013 ; PA : Z=4.04, voxel= 28, p=.04<br>**2.** CTQ Total : Z=3.54, voxel= 41, p=.021<br>PN : Right Thalamus, Z=4.25, voxel= 321, p=.002<br>Left thalamus Z=3.30, voxel=15, p=.04<br>EN: Right thalamus Z=3.31, voxel=29, p=.04<br>(All results show a negative association)	Age<br>Sex<br>Total GMV	no
**Du Plessis et al. (2020)**	FEP	DSM-IV	CTQ Composite (continuous)	1.Hippocampus	**1.** NSA	Age<br>Sex<br>Diagnosis<br>Scanner-Sequence<br>ICV<br>Substance Abuse	no
**Elvsåshagen et al. (2013)**	BD II	DSM-IV	CTQ composite (continuous)	Hippocampus subfields<br>1.Left DG–CA4<br>2. Right DG–CA4<br>3. Left CA2–3	1. NSA<br>2. NSA<br>3. NSA	Total GMV<br>Age<br>Group status	no
**Fan et al. (2022)**	SCZ	DSM-IV	CTQ composite and subtypes<br><br>(continuous)	1.Amygdala<br>2.Thalamus<br>3.Caudate<br>4.Putamen<br>5.Pallidum<br>5.Nucleus accumbens<br>6.Hippocampus<br>7.Total cortical GMV	1.r =-0.20, p = 0.03<br>2.NSA<br>3.NSA<br>4.NSA<br>5.NSA<br>6.NSA<br>7.NSA	Age<br>Sex<br>ICV<br>Bonferroni correction	yes
**Frissen et al. (2018)**	SCZ	DSM-IV	CTQ Composite<br><br>(continuous)	1.Total GMV	**1.**B = -9.79, *p* = .01	Age<br>Sex<br>Educational level<br>ICV<br>Scan type	no
**Harmata et al. (2023)**	BD-I	SCID<br>DSM-V<br>BD-I	ACE score	1.Left cerebellar GMV<br>2.Right cerebellar GMV<br>3.Vermis I-V	1.p=0.019, q=0.033<br>2.p=0.018, q=0.033<br>3.NSA	Age<br>Sex<br>ICV	no

Supplementary material

				4.Vermis VI-VII<br>5.Vermis VII-X	4.NSA<br>5.NSA	FDR correction	
**Hernaus et al. (2014)**	SCZ	DSM-IV	CTQ<br>Composite<br>(continuous)	1.Left Hippocampus<br>2.Right Hippocampus	**1.** NSA<br>**2** .NSA	ICV<br>Age<br>Gender<br>Educational level<br>Drug use	yes
**Hoffmann et al. (2018)**	SCZ<br>SZA	DSM-IV	CAQ<br>Composite &<br>Subtypes: PA, SA, EN, EA, loss, family dysfunction, financial dysfunction<br>(continuous)	1. Caudate<br>2. Putamen<br>3. Nucleus Accumbens<br>4. DLPFC<br>5.Hippocampus	**1.** Right Caudate, PA: B =16.00, 95% CI = 44.00-26.00, p = 0.008.<br>**2**. NSA<br>**3.** NSA<br>**4.** NSA<br>**5**. NSA	Age<br>Gender<br>Scanning site<br>ICV	no
**Hoy et al. (2011)**	FEP	ICD 10	TEC<br>Composite<br>(Binary)	1. Total GMV, WM, CFS & ICV<br>2. Hippocampus<br>3. Amygdala<br>4. Hippocampus & Amygdala Complex	**1.** NSA<br>**2.** NSA<br>Right hippocampus: NSA<br>Left hippocampus: β = -.40, *p* =.03<br>**3.** β = -0.5, p = 0.019<br>Right Amygdala: β = -.42, p = .04<br>Left Amygdala: NSA<br>**4.** β = .45, *p* = .03	Age<br>Sex<br>Delay to scan time	yes
**Janiri et al., (2017)**	BD-I<br>BD-II	DSM-IV<br>BD-I<br>BD-II	CTQ<br>Composite<br>(continuous)	1. Amygdala<br>2.Hippocampus	1.Right (BD-I) (t=2.88, p=.006)<br>Right (BD-II) NSA<br>Left (BD-I) (t=1.54, p=.012)<br>Left (BD-II) (2.54, p=.01)<br>2.Right (BD-I) NSA<br>Right (BD-II) (t=2.27, p=.02)<br>Left (BD-I) NSA<br>Left (BD-II) (t=2.54, p=.01)<br>(results indicate bigger volume in patients in relation to CA)	ICV<br>Sex	yes
**Janiri et al., (2019)**	BD-I<br>BD-II	DSM-IV	CTQ<br>Composite<br>(continuous)	Hippocampal subfield volume<br>1.Right CA1	**1.**t=1.12, .26 (BD-I)<br>t=1.70, p=.09 (BD-II)<br>**2.** t=2.28, .02 (BD-I)	DUI	no

Supplementary material

				2. Right Presubiculum<br>3. Right subiculum<br>4.Left CA1<br>5. Left Presubiculum<br>6.Left subiculum	t=3.11, p=.003 (BD-II)<br>**3.** t=2.28, .02 (BD-I)<br>t=3.11, p=.003 (BD-II)<br>**4.**t=.66, .50 (BD-I)<br>t=2.35, p=.002 (BD-II)<br>**5.**t=1.65, .10 (BD-I)<br>t=3.37, p=.001 (BD-II)<br>**6.**t=2.02 .04 (BD-I)<br>t=2.75, p=.008 (BD-II)<br>(results indicate bigger volume in relation to CA)		
**Millman et al. (2022)**	SCZ	DSM-IV-TR (SCID)	CTQ	1.Left and right amygdala<br>2.Left and right hippocampus	**1.**Left amygdala: r = 0.30, p=0.01<br>Right amydala NSA<br>**2.**Left hippocampus: NSA<br>Right hippocampus: NSA	Sex<br>ICV	yes
**Poletti et al., (2014)**	BD-I	DSM-IV	RFQ Composite (continuous)		**1.** MNI coordinates −33 −15 -21 ; T = 3.53, Z = 3.38, p FWE = .02<br>(results indicate a volume reduction in relation to CA)	Yes<br>Age<br>Sex<br>DUI<br>No.of eps.<br>Handedness	no
**Quidé et al. (2021)**	SCZ<br>SZA<br>BD	ICD-10	CTQ Composite (continuous), High, average and low trauma exposure	1. IC2:<br>Precuneus, Cingulate Gyrus, Inferior Parietal Lobule, Posterior Cingulate, Superior Parietal Lobule<br>2.IC6:<br>Declive, Tuber, Lentiform Nucleus, Culmen, Uvula, Cerebellar Tonsil, Pyramis, Caudate, Fusiform Gyrus	SCZ, SZA<br>**1**. t = 2.064, p = .041 (low trauma)<br>**2**. NSA<br><br>BD<br>1.NSA<br>2.NSA	Age<br>Sex<br>ICV<br>Group status<br>Bonferroni adjustments	no
**Rokita et al. (2020)**	SCZ<br>SZA	DSM-IV	CTQ Composite & PN (Continuous)	1.Amygdala<br>2.Hippocampus<br>3.ACC	**1**.NSA<br>**2**.NSA<br>**3**.r = -0.161, p = 0.048	Age<br>Sex<br>Years of Education<br>ICV	no

Supplementary material

**Ruby et al. (2017)**	SCZ	DIGS	Early Trauma Inventory Composite (Continuous)	1.Whole Brain volume<br>2.Amygdala/Whole Brain Volume Ratio<br>3.Hippocampal/Amydala Volume Ratio<br>4.Hippocmapus/Whole Brain Volume Ratio	**1**.r = -.50, (n = 17), *p* = .04<br>**2**.r = .56, (n = 17), *p* = .02<br>**3**.NSA<br>**4**.NSA	-	yes
**Sheffield et al. (2013)**	SCZ<br>SZA<br>BP-I	DSM-IV	CTQ Composite & Subtypes: PA, SA, EA, EN, PN (Binary)	1.total GMV<br>2. 7 regions within the PFC<br>3. Hippocampus<br>4. Amygdala	**1**.Total CTQ (r = -.27, *p* = .04)<br>SA (r = -.34, *p* = .008)<br>NSA PA,EA, EN, PN,<br>**2**. Left middle frontal gyrus (k = 265, -48, 23, 31), p < 0.005<br>**3.** NSA<br>**4.** NSA	Age<br>Sex	no
**Song et al., (2020)**	BD-I<br>BD-II	DSM-V	CTQ Composite (Continuous)	1.Right precentral gyrus<br>2.Left Middle Frontal Gyrus	1.r=-.61, *p*<.0003<br>2.NSA	Age<br>Sex<br>ICV	no
**Souza-Queiroz et al., (2016)**	BD	DSM-IV	CTQ Composite Subtypes (EN/PN) (continuous)	1.Hippocampus<br>2.Amygdala	**1.** NSA<br>**2**. β = -0.22, p = .04 (HC and BD), BD only NSA	Yes<br>Age<br>Gender<br>Education<br>ICV<br>DUI<br>Medication	no
**Tseng et al. (2021)**	SCZ	DSM-IV	BBTS Composite (Continuous)	1. Thalamus<br>2. Inferior Fontal Gyrus<br>3. Insula<br>4. Superior Frontal Gyrus<br>5. Precuneus	**1.**NSA<br>**2.** NSA<br>**3.** NSA<br>**4.** NSA<br>**5.** NSA	DUI<br>Medication	no

Supplementary material

Supplementary table 6. Summary of meta-regressions

Meta-analysis	Hemisphere	Moderator	β	95% CI	*p*-value	Heterogeneity (I^2)	Between study variance (τ^2)
Hippocampus	Left	Age	0.010	-0.016; 0.037	0.449	51.48%	0.021
		Sex	0.002	-0.020; 0.023	0.871	62.26%	0.026
		Illness stage	0.061	-0.297; 0.419	0.738	55.45%	0.022
		Segmentation Method	-0.081	-0.487; 0.325	0.696	62.7%	0.025
		Study Quality (NOS)	0.217	-0.055; 0.491	0.118	29.43%	0.007
	Right	Age	0.008	-0.016; 0.033	0.495	46.11%	0.016
		Sex	-0.004	-0.023; 0.0157	0.708	49.95%	0.015
		Illness stage	0.202	-0.042; 0.445	0.105	41.46%	0.012
		Segmentation Method	-0.049	-0.428; 0.330	0.801	41.46%	0.019
		Study Quality (NOS)	0.242	0.023; 0.461	**0.030**	41.46%	0.011
	Total	Age	0.011	-0.016; 0.038	0.443	60.16%	0.031
		Sex	0.020	0.007; 0.035	**0.007**	7.31%	0.001
		Illness stage	0.125	-0.271; 0.520	0.537	59.77%	0.023
		Segmentation Method	-0.281	-0.583; 0.022	0.068	42.63%	0.014
		Study Quality (NOS)	0.230	0.042; 0.418	**0.016**	25.60%	0.006
Amygdala	Left	Age	0.016	-0.046; 0.078	0.614	85.5%	0.069
		Sex	-0.013	0.026; 0.037	0.611	85.83%	0.102
		Illness stage	0.513	0.199; 0.122	**0.01**	47.66%	0.016
		Segmentation method	-0.516	-0.903; -0.991	**0.01**	47.66%	0.016
		Study Quality (NOS)	0.513	0.199; 0.122	**0.01**	47.66%	0.016
	Right	Age	0.018	-0.017; 0.054	0.318	60.09%	0.036
		Sex	-0.005	-0.044; 0.034	0.798	73.12%	0.046
		Illness stage	0.378	0.085; 0.671	**0.011**	0%	0.000
		Segmentation method	-0.378	-0.671; -0.085	**0.011**	0%	0.000
		Study Quality (NOS)	0.378	0.08; 0.671	**0.011**	0%	0.000
	Total	Age	0.010	-0.039; 0.059	0.689	85.76%	0.113
		Sex	0.001	-0.044; 0.046	0.958	83.41%	0.090
		Illness stage	0.603	0.123; 1.083	**0.014**	82.58%	0.082
		Segmentation method	-0.202	-0.743; 0.339	0.465	83.84%	0.086
		Study Quality (NOS)	-0.072	-0.471; 0.327	0.098	85.92%	0.098

Supplementary material

Supplementary Figure 1a

Forest plot of association between left hippocampal volume and CA

Study name	Correlation	Lower limit	Upper limit	Z-Value	p-Value
Aas et al. (2012)	0.100	-0.199	0.382	0.650	0.516
Hernaus et al. (2014)	-0.200	-0.392	0.009	-1.880	0.060
Hoy et al. (2011)	-0.400	-0.709	0.038	-1.797	0.072
Janiri et al. (2017)	0.090	-0.103	0.277	0.911	0.362
Millman et al. (2022)	0.060	-0.165	0.279	0.520	0.603
Pooled	-0.036	-0.196	0.127	-0.427	0.669
Prediction Interval	-0.036	-0.483	0.427		

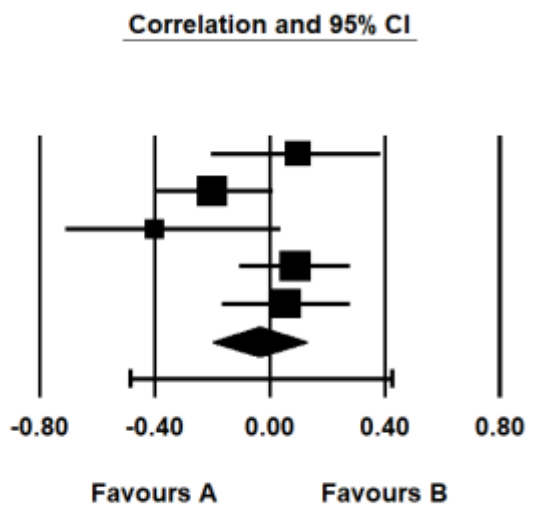

Meta Analysis

Supplementary Figure 1b

Forest plot of association between right hippocampal volume and CA

Study name	Correlation	Lower limit	Upper limit	Z-Value	p-Value
Aas et al. (2012)	0.050	-0.247	0.339	0.324	0.746
Hernaus et al. (2014)	-0.190	-0.383	0.019	-1.784	0.074
Hoy et al. (2011)	-0.160	-0.553	0.292	-0.685	0.494
Janiri et al. (2017)	0.080	-0.113	0.268	0.810	0.418
Millman et al. (2022)	0.180	-0.044	0.387	1.576	0.115
Pooled	0.010	-0.138	0.158	0.135	0.893
Prediction Interval	0.010	-0.388	0.405		

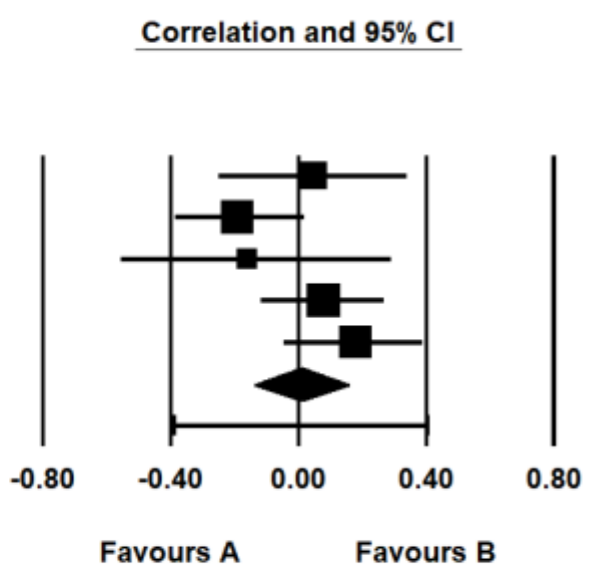

Meta Analysis

Supplementary material

Supplementary Figure 2a

Forest plot of association between left amygdala volume and CA

Study name	Statistics for each study					Correlation and 95% CI
	Correlation	Lower limit	Upper limit	Z-Value	p-Value	
Aas et al. (2012)	-0.150	-0.425	0.150	-0.980	0.327	
Hoy et al. (2011)	-0.520	-0.777	-0.114	-2.445	0.014	
Janiri et al. (2017)	0.120	-0.073	0.305	1.218	0.223	
Millman et al. (2022)	0.300	0.083	0.490	2.681	0.007	
Pooled	-0.022	-0.309	0.268	-0.147	0.883	
Prediction Interval	-0.022	-0.869	0.858			

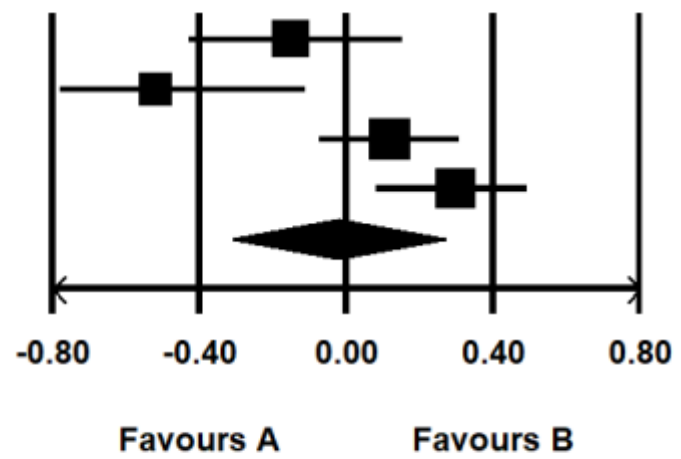

Meta Analysis

Supplementary Figure 2b

Forest plot of association between right amygdala volume and CA

Study name	Statistics for each study					Correlation and 95% CI
	Correlation	Lower limit	Upper limit	Z-Value	p-Value	
Aas et al. (2012)	-0.150	-0.425	0.150	-0.980	0.327	
Hoy et al. (2011)	-0.420	-0.721	0.014	-1.899	0.058	
Janiri et al. (2017)	0.150	-0.043	0.332	1.526	0.127	
Millman et al. (2022)	0.120	-0.105	0.334	1.044	0.296	
Pooled	-0.017	-0.230	0.198	-0.153	0.878	
Prediction Interval	-0.017	-0.709	0.692			

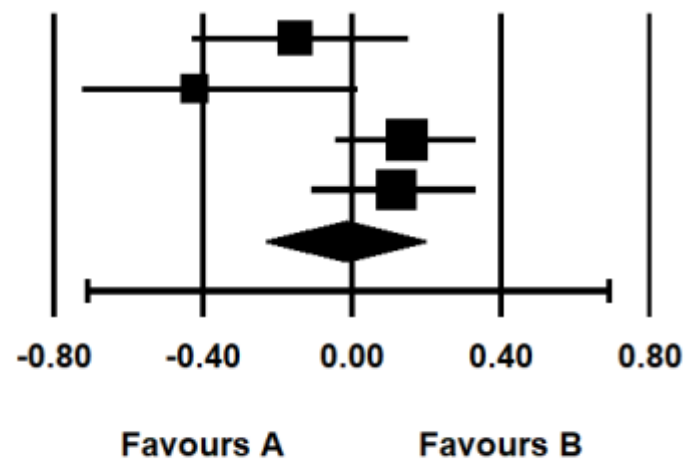

Meta Analysis

Supplementary material

Supplementary Figure 3a

Forrest plot of association between total hippocampal volume and CA

Study name	Correlation	Lower limit	Upper limit	Z-Value	p-Value
Aas et al. (2012)	-0.150	-0.425	0.150	-0.980	0.327
Fan et al. (2023)	-0.187	-0.350	-0.013	-2.107	0.035
Hernaus et al. (2014)	-0.190	-0.575	0.263	-0.816	0.414
Hoy et al. (2011)	-0.315	-0.657	0.135	-1.383	0.167
Janiri et al. (2017)	0.085	-0.108	0.272	0.861	0.389
Millman et al. (2022)	0.120	-0.105	0.334	1.044	0.296
Ruby et al. (2018)	-0.470	-0.717	-0.118	-2.550	0.011
Pooled	-0.118	-0.271	0.040	-1.462	0.144
Prediction Interval	-0.118	-0.510	0.315		

Statistics for each study

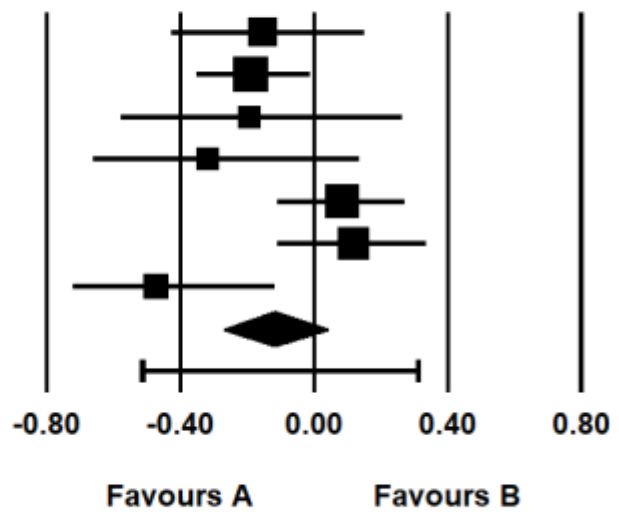

Meta Analysis

Supplementary Figure 3b

Forrest plot of association between total amygdala volume and CA

Study name	Correlation	Lower limit	Upper limit	Z-Value	p-Value
Aas et al. (2012)	-0.400	-0.621	-0.121	-2.746	0.006
Fan et al. (2023)	-0.200	-0.362	-0.027	-2.258	0.024
Hoy et al. (2011)	-0.500	-0.766	-0.087	-2.331	0.020
Janiri et al. (2017)	0.130	-0.063	0.314	1.320	0.187
Millman et al. (2022)	0.210	-0.013	0.413	1.846	0.065
Ruby et al. (2017)	0.560	0.126	0.814	2.451	0.014
Pooled	-0.042	-0.296	0.218	-0.310	0.756
Prediction Interval	-0.042	-0.727	0.686		

Statistics for each study

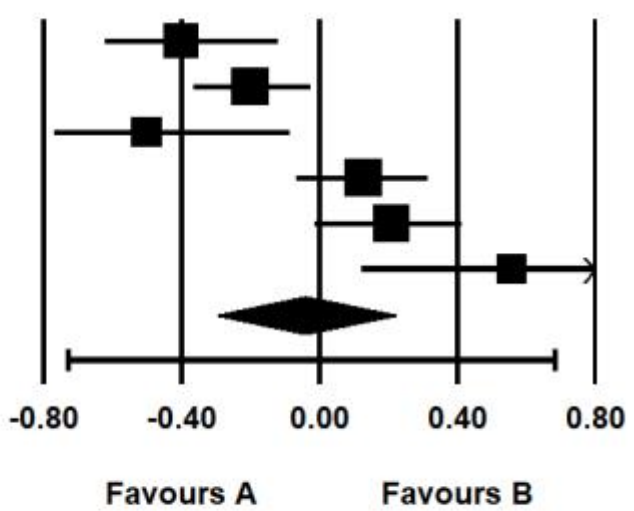

Meta Analysis
